# Caregiving and mental health needs in the significant others of women receiving inpatient and home treatment for acute severe postpartum mental illness

**DOI:** 10.1007/s00737-022-01286-w

**Published:** 2022-12-24

**Authors:** Katie H. Atmore, Billie Lever Taylor, Laura C. Potts, Kylee Trevillion, Louise M. Howard

**Affiliations:** 1 Section of Women’s Mental Health, Institute of Psychiatry Psychology and Neuroscience, King’s College London, London, UK; 2 Department of Biostatistics and Health Informatics, Institute of Psychiatry, Psychology and Neuroscience, King’s College London, London, UK

**Keywords:** Significant others, Perinatal, Parental mental health, Caregiving, Crisis care, Inpatient

## Abstract

To examine the mental health and caregiving needs of significant others (including partners, parents, friends) to women who received acute psychiatric care either as inpatients or at home during the perinatal period. Cross-sectional survey of 98 significant others of 279 women who participated in a quasi-experimental cohort study of services for mothers with acute severe postpartum mental health diagnoses. Significant others completed an adapted General Health Questionnaire-12 (GHQ) and Involvement Evaluation Questionnaire (IEQ) to indicate their mental health needs and service use as well as caregiving activities. The mean age of significant others was 38.9 years (range 24–69). 81.6% were male and 81.6% were intimate partners to the women. High levels of unmet mental health needs were detected in significant others, with a majority (51.0%) having a score > 2 on the GHQ-12 indicating caseness for a psychiatric disorder. In those with GHQ-12 caseness indicated, few were receiving help for their difficulties: 22.5% received support from their general practitioner, and 14.3% received help from a social worker, psychologist, psychiatrist or outpatient department. 18.4% received medication for GHQ-12 symptoms. The median sumscore of IEQ surveying caregiving activities in significant others was 18/108. We did not find evidence of differences in GHQ-12 or IEQ scores for significant others to women who received inpatient care versus care at home. Significant others to women with acute severe postpartum psychiatric illness have high levels of unmet mental health needs during the weeks after women are discharged from acute care. Services need to address these needs to optimise outcomes for the whole family.

## Introduction

Most research and service design in perinatal mental health has focused on mothers. However, in the United Kingdom (UK), the National Health Service (NHS) Long Term Plan highlights the need to offer *‘fathers/partners of women accessing specialist perinatal mental health services and maternity outreach clinics evidence-based assessment for their mental health and signposting to support as required’*. Internationally, there are calls for perinatal mental health care to be more inclusive of partners (Fisher et al. 2021) and other family members supporting mothers and infants (Perera et al. 2014). Assessment methods for identifying mental illness in partners have recently been reviewed (Darwin et al. 2020) and, in the UK, new best practice guidance has been published on supporting and involving partners in perinatal mental health services (Darwin et al. 2021). However, there have been few studies quantifying levels of mental health needs in significant others, even though this is essential for planning services to improve psychological provision.

Two meta-syntheses provide an overview of qualitative studies exploring the experiences of significant others to women with postpartum psychiatric needs. The first identified 20 studies about how partners viewed perinatal mental health care and noted that, although partners were often facing struggles themselves, they did not find perinatal services to be inclusive of their needs. They had a limited understanding of how to seek help for themselves and were concerned that they would be judged for opening up to services (Lever Taylor et al. 2018). The other meta-synthesis included 15 studies about experiences of recovery after postpartum psychosis from the perspectives of women and family members. It included the views of 42 family members and argued that service providers need to offer more collaboration and inclusion of the wider family to enhance family relationships and ways of coping (Forde et al. 2020). In a study of particular relevance to the present analysis, women receiving acute psychiatric perinatal care in a variety of settings and their significant others were interviewed (Lever Taylor et al. 2019). Significant others were found to be a critical influence on women’s mental health, and described significant mental distress themselves, but reported that they felt marginalised by services and cautious about what being included by services might involve.

**Fig. 1:**
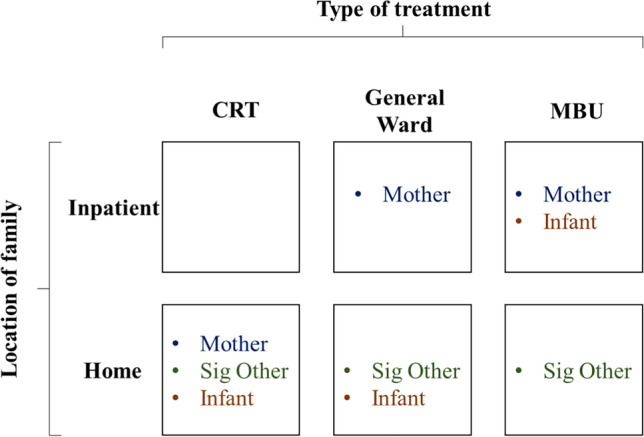
Perinatal mental health care settings in the UK

Quantitative data on this topic is limited. In one UK study, 50% of partners to mothers with acute psychiatric needs treated in inpatient mother and baby units (MBUs) met the criteria for a psychiatric disorder themselves according to Research Diagnostic Criteria (Lovestone and Kumar 1993). Another UK MBU study found that 42% of partners to women admitted for postpartum psychiatric care had psychiatric ‘morbidity’ when interviewed using the Psychiatric Assessment Schedule (Harvey and McGrath 1988). These studies focused on partners of mothers accessing specialist MBUs, though in practice, perinatal acute pathways also include generic, non-specialist services, such as general psychiatric wards (the main location of acute care internationally) and crisis resolution teams (CRT). CRTs have been introduced in countries including the UK, Australia, Norway, Belgium, France, and the USA (Johnson 2013) and deliver intensive care at home as an alternative (or in addition) to hospital care (Fig. 1). Research indicates that some mothers prefer to receive acute treatment at home rather than as an inpatient (Khalifeh et al. 2009). Some women also report difficulties with intensive home treatment in the perinatal period, because of a lack of specialist perinatal expertise and tailoring to the perinatal context (Rubio et al. 2021).

The needs of significant others may also vary depending on the type of care that mothers receive. Qualitative research has reported that significant others feel most supported by MBUs compared to other services and that significant others to mothers receiving care at home may need additional support in-between visits from CRT staff (Lever Taylor et al. 2019).

At the time of publishing, no studies have quantitatively explored the needs of significant others (including partners and others who provide support such as grandparents) to women receiving acute care in the perinatal period across different types of settings, beyond only MBUs. This analysis, therefore, investigated mental health needs and service use as well as caregiving activities in significant others to women treated at home by CRTs, and/or as inpatients on MBUs or general psychiatric wards to provide vital information for service planning.

## Materials and methods

### Recruitment

This data was collected as part of the ESMI MBU study, a quasi-experimental cohort study of women receiving different types of acute psychiatric care up to 1-year post-partum in the NHS. A comprehensive methodology of the original study can be found in the published protocol (Trevillion et al. 2019). Women were included if they received care from at least one of the following: MBU, general psychiatric ward, or CRT. In the UK, MBUs are inpatient units with a minimum of four beds delivering acute psychiatric care to mothers and infants and separated from other ward types (Elkin et al. 2009). General psychiatric wards are inpatient psychiatric wards and do not provide care for infants. Community treatment encompasses CRTs and home treatment teams which provide care for those in acute psychiatric crisis at home with 24 hour accessibility (Wheeler et al. 2015).

To be included, women needed to have been admitted to services between March 2013 and December 2017 and have the capacity to consent. There were no language exclusions and interpreters were used where necessary. Women were excluded if the admission was prophylactic (i.e. services were solely for monitoring purposes) or if their baby had been removed from their care before admission to services. The full results and final report of the main ESMI MBU study, providing in-depth information about the women involved in the study, have also been published elsewhere (Howard et al. 2022a, b). Ethical permission was granted by the NHS Research Ethics Committee (London-Camberwell St. Giles): 14/LO/0765.

### Survey of significant others

During face-to-face interviews, at least 1 month after discharge from acute services, women were asked to identify a ‘significant other’, defined as someone who supported them whilst they were under the care of services. Significant others were approached by the research team regarding participation and if, after learning about the study, they consented to take part they completed a paper or online survey including demographic characteristics and two outcome questionnaires relating to mental health, service use, and caregiving activities. Participants were thanked for their time with a £10 shopping voucher. The demographic questions included gender, age, civil status, relationship to mother receiving treatment, household occupants, income, and education. The outcomes were:

1. GHQ-12: main outcome
  A widely used rapid 12-item screening tool to assess for psychiatric disorders (Goldberg 1988). Standard dichotomous GHQ-12 scoring to present total scores and to indicate caseness for psychiatric disorders was used. Each item has four possible responses (0= Not at all, 0= Not more than usual, 1= More than usual, 1=Much more than usual). Total scores are given out of a possible total of 12, with scores of 3 and above indicating caseness for psychiatric disorders (Trevillion et al. 2019). Three yes/no questions were added to understand mental health service usage: ‘*Do you receive help from your GP for any GHQ-12 complaints?, Do you receive help from a social worker, psychologist, psychiatrist, or outpatient department for any GHQ-12 complaints, Are you taking medicine for any GHQ-12 complaints?*’.
2. IEQ: secondary outcome
  The IEQ quantifies the experiences of individuals who are close to someone with (mental) health needs. The IEQ is scored on 27 questions relating to the significant other’s relationship to the mother receiving psychiatric care in the previous 4 weeks and the options for answering are never (0), sometimes (1), regularly (2), often (3), and (almost) always (4) which are scored on a Likert scale (Schene et al. 2006). The sumscore is calculated out of a total of 108. The 27 IEQ questions are divided into subscales for ‘Urging’, e.g. *How often have you encouraged your relative/friend to eat enough?* (/36); ‘Supervision’, e.g. *How often have you ensured that your friend/relative received sufficient sleep?* (/24); ‘Tension’, e.g. *How often has the atmosphere been strained between you both, as a result of your relative/friend’s behaviour?* (/24); and ‘Worrying’, e.g. *How often have you worried about your relative/friend’s safety?* (/32). The IEQ has been validated and recommended as the most comprehensive tool for measuring outcomes of caregivers to populations with mental illness (van Wijngaarden et al. 2002).

### Statistical analysis

Descriptive statistics were used to summarise the data. Categorical measures were described using totals and percentages. Continuous symmetric (non-skewed) measures were described using mean and standard deviation, whilst skewed measures were described using median and ranges.

In line with the original study design, data are split by service type received by the mother. Data from significant others to women receiving inpatient care from either MBUs or general psychiatric wards were combined due to low numbers of participant responses and because these groups were defined in the original study as having higher levels of care. Data were then analysed based on whether women had received inpatient care or CRT care, with the null hypothesis that there was no difference between these groups. If women received both inpatient and CRT care, significant others were allocated to the inpatient group. Differences between demographic and outcomes by service type were explored using chi-squared tests for proportions and Mann–Whitney tests for continuous (non-parametric) data. Full test results for differences across demographics were only presented where significant. Data indicating service use by significant others were split by GHQ-12 caseness to maximise the clinical utility of the present study. All analyses were conducted in STATA 17.0.

## Results

Of the 279 mothers who participated in the ESMI MBU evaluation, 83.9% (*n* = 234) consented to a significant other being contacted and, of these, 41.9% (*n* = 98), significant others consented to the study and completed the questionnaire. Characteristics of significant others are indicated in Table 1. Forty-four were significant others to the 109 women who received CRT care only and 54 were significant others to the 170 women who received inpatient care. The inpatient care group was comprised of 33 significant others to the 108 women who received MBU services and 21 significant others to the 62 women who were treated in general psychiatric wards. Response rates did not significantly differ between groups.

**Table 1:** Demographic characteristics of significant others

*n* (%)	Significant others to women receiving CRT care only 44 (44.9)	Significant others to women receiving inpatient care from general psychiatric ward/MBU 54 (55.1)	Totals 98
Response rate, *n* (%)			
Number of significant other responses/number of women asked to identify significant other	**44/109** (40.4)	**54/170** (31.8)	**98/279** (35.1)
Gender, *n* (%)			
Male Female	**38** (86.4) **6** (13.6)	**42** (77.8) **12** (22.2)	**80** (81.6) **18** (18.4)
Age years, *n*	**43**	**51**	**94**
Mean, standard deviation, range	38.2, 10.3, 24–67	39.5, 9.8, 25–69	38.9, 10.0, 24–69
Civil status, *n* (%)			
Married/partner Single/divorced/widowed	**42** (95.5) **2** (4.5)	**52** (94.4) **3** (5.6)	**93** (94.9) **5** (5.1)
Relationship to mother, *n* (%)			
Partner Parent Other (child/sibling/friend)	**36** (81.8) **6** (13.6) **2** (4.6)	**44** (81.5) **8** (14.8) **2** (3.7)	**80** (81.6) **14** (14.3) **4** (4.1)
Living with mother, *n* (%)			**97**
Yes No	**40** (90.9) **4** (9.1)	**49** (92.4) **4** (7.6)	**89** (91.8) **8** (8.3)
Significant other family income per month, *n* (%)			**96**
Up to £900 £900–£1500 £1500–£2250 £2250 + Rather not say	**2** (4.6) **11** (25.0) **15** (34.1) **14** (31.8) **2** (4.6)	**11** (21.2) **3** (5.8) **11** (21.2) **22** (42.3) **5** (9.6)	**13** (13.5) **14** (14.6) **26** (27.1) **36** (37.5) **7** (7.3)
1st episode of psychiatric disorder in mother, *n* (%)			
Yes No	**15** (34.1) **29** (65.9)	**22** (40.7) **32** (59.3)	**37** (37.8) **61** (62.2)

*n* is indicated in bold and presented in totals column where missing data occurs; percentages are shown in brackets

Most significant others were male (*n* = 80, 81.6%), and the same number were either married to or a partner to the mother (*n* = 80, 81.6%). These corresponding proportions are not indicative that all males were partners and all partners were male. Some males were parents of mothers in the study and some partners were female. The mean age was 38.9 years (SD = 10.0). Significant others predominately lived with the mother (*n* = 89, 91.8%). More than a third of significant others reported levels of income greater than £2500 per month (*n* = 37, 37.5%) though 7 participants (7.3%) declined to answer. For the majority of women, there was a history of previous psychiatric episodes (*n* = 61, 62.2%). There were no statistically significant differences in any of these demographic characteristics between inpatient and CRT groups.

Forty-nine (51.0%) significant others had a score of 3 or more on the GHQ-12 indicating probable psychiatric caseness. The median total GHQ-12 score was 3/12 and the interquartile range (IQR) was 0–6 (Table 2). Of those who were indicated as having psychiatric caseness on the GHQ-12, only 11 (22.5%) were receiving help from their general practitioner (GP) for any of their GHQ-12 complaints, and only 7 (14.3%) received any help from a social worker, psychologist, psychiatrist, or outpatient department for their complaints and only 9 (18.4%) were receiving medication for GHQ-12 symptoms (Table 3).

**Table 2:** Results of survey

*n* (%)	Significant others to women receiving CRT care only 44 (44.9)	Significant others to women receiving inpatient care from general psychiatric ward/MBU 54 (55.1)	Totals 98	Test statistic and *p*-value
GHQ-12 caseness *n* (%)			**96**	
Yes No	**19** (44.2) **24** (55.8)	**30** (56.6) **23** (43.4)	**49** (51.0) **47** (49.0)	*Χ*^2^ = 1.4649 *p* = 0.226
GHQ-12 total score/12 *n*	**43**	**53**	**96**	*z* = − 0.709
Median, IQR, range	2, 0–6, 0–12	3, 1–6, 0–12	3, 0–6, 0–12	*p* = 0.4781
IEQ sumscore/108 *n*	**41**	**49**	**90**	*z* = − 0.981
Median, IQR, range	18, 11–29, 2–56	23, 12–37,1–61	19, 11–32, 1–61	*p* = 0.3267
IEQ — urging/36 *n*	**43**	**51**	**94**	*z* = − 0.958
Median, IQR, range	6, 4–11, 0–24	8, 3–13, 0–23	7, 4–12, 0–24	*p* = 0.3379
IEQ — supervision/24 *n*	**42**	**49**	**91**	*z* = − 0.976
Median, IQR, range	2, 1–4, 0–6	3, 2–5, 0–13	2, 1–2, 0–13	*p* = 0.3293
IEQ — tension/24 *n*	**43**	**51**	**94**	*z* = − 0.700
Median, IQR, range	6, 3–9, 0–28	7, 4–9, 0–18	6, 3–9, 0–28	*p* = 0.4837
IEQ — worrying/32 *n*	**44**	**53**	**97**	*z* = − 1.171
Median, IQR, range	4.5, 2.5–8.5, 0–18	8, 3–12, 0–19	6, 3–11, 0–19	*p* = 0.2416

*n* is indicated in bold and presented in totals column where missing data occurs; percentages are shown in brackets. Statistical tests conducted were chi-squared test for proportions and Mann–Whitney test for continuous (non-parametric) data

**Table 3:** Unmet mental health needs

*n* (%)	GHQ-12 non-case 47 (49.0)	GHQ-12 case 49 (51.0)	Totals 96
Do you receive help from your GP for any GHQ-12 complaints? *n* (%)			**95**
Yes No	**5** (10.9) **41** (89.1)	**11** (22.5) **38** (77.6)	**16** (16.8) **79** (83.2)
Do you receive help from a social worker, psychologist, psychiatrist, or outpatient department for any GHQ-12 complaints? *n* (%)			**96**
Yes No	**2** (4.3) **45** (95.7)	**7** (14.3) **42** (85.7)	**9** (9.4) **87** (90.6)
Are you taking medicine for any GHQ-12 complaints? *n* (%)			**96**
Yes No	**3** (6.4) **44** (93.6)	**9** (18.4) **40** (81.6)	**12** (12.5) **84** (87.5)

*n* is indicated in bold and presented in totals column where missing data occurs; percentages are shown in brackets

The median sumscore of the IEQ for significant others (*n* = 90) was 19 (out of a possible 108) with an IQR of 11–32 (Table 2). Of the 27 IEQ items, 10 had mean scores > 1 indicating that significant others spent comparatively more time on these aspects of caregiving (more often than ‘sometimes’): encouraging eating/sleeping/other activities in the mother, ensuring medication compliance, carrying out tasks normally done by mother, worrying about the mother’s general health/finances/future, worrying about their own future, and feeling the mother’s mental health had been a burden.

There was no evidence of a difference in GHQ-12 totals, caseness, or IEQ sumscore/subscale scores between significant others to mothers receiving CRT care only and significant others to mothers receiving inpatient care.

## Discussion

### Key findings

Fifty-one percent of significant others to women accessing acute postpartum psychiatric care had GHQ-12 ‘caseness’, supporting previous findings (Harvey and McGrath 1988; Lovestone and Kumar 1993) that there are high levels of mental health need in this population. This survey was the first of its kind to include data from multiple settings, including CRTs and inpatient general psychiatric wards as well as MBUs. Our analysis did not find evidence of differences in significant others’ reported mental health needs according to the setting where women were treated. This study is the first to ascertain whether significant others were receiving help for their mental health needs and the results indicate that this population has high levels of unmet needs. Around 80% of participants who had GHQ-12 scores high enough to indicate caseness received no support for their mental health symptoms from GPs, social workers, mental health specialists, or outpatient departments and were not taking any medication for these complaints.

We also looked at the caregiving activities of significant others using the IEQ. The median IEQ sumscore for significant others in this study (19) was higher than that reported for carers to individuals with substance use conditions (18.2) (Kronenberg et al. 2016). However, it was lower than the median IEQ score for significant others to those receiving inpatient care after a suicide attempt (23.5) (Magne-Ingvar and Öjehagen 2005) or those with a diagnosis of schizophrenia (23.6) (van Wijngaarden et al. 2000). Though the IEQ is not a clinical measure, it does help to indicate the level of caring needs of significant others to service providers. Despite this, the IEQ is not well suited to capture specific perinatal needs such as childcare which are likely to be elevated among significant others in this context.

### Limitations

The core limitation of this dataset is that the responding sample was small, reducing the power of our analyses and meaning that we had to group MBU and general psychiatric ward data. There were no differences found in the present analysis between significant others to mothers who received either inpatient treatment or CRT care at home. Larger samples of the three separate groups who have had family units split up in various ways (Fig. 1) may go further in elucidating the specific needs of these groups as well as mechanisms underlying poor mental health. Significant others living with mothers receiving CRT care may well have additional stressors in caring for mothers as well as caring for infants at home (Khalifeh et al. 2009) and may also be working or unable to take parental leave. Significant others who remain at home whilst mothers are treated in MBUs along with their infants report feeling unsupported and needing to stay strong despite struggling themselves (Boddy et al. 2017) as well as difficulties in bonding with infants and travelling to MBUs (Marrs et al. 2014). There is currently little data on the experiences of significant others who care for infants at home whilst mothers are on general psychiatric wards, and whether their experience is similar to significant others to women in MBUs who are admitted with their infants. Women with higher levels of acute psychiatric postpartum need are more likely to be admitted for inpatient care. This may lead to higher levels of stress in significant others especially if these admissions involve sectioning under the mental health act. In addition, MBUs are not available in many countries and score higher on service satisfaction metrics than other acute mental health service types for women during the perinatal period (Howard et al. 2022b).

Another consideration is that significant others may be more likely to respond to requests to complete the survey if they have more stable circumstances or mental health and find it easier to participate in research. Significant others with very poor mental health may not have completed the survey. Of the total 279 mothers included in the ESMI-II study, only 98 significant others completed the survey so there may be differing levels of mental health needs in the significant others who did not respond to requests to complete the survey.

Finally, the present questionnaires did not collect data on the mental health status of significant others before, or after the acute, severe perinatal mental illness of the mother, and this data would strengthen the understanding of significant others’ mental health over time.

### Future research

Though the number of participants who were not male partners to women represented a minority of the data, it is critical to highlight the needs of all caregivers in a woman’s social network and to ensure that services use broad definitions of significant others beyond just fathers. Future work could include larger samples of significant others including grandparents, same-sex partners, and other household members, e.g. other children to ascertain if rates of mental health symptoms differ between groups. In the absence of data on the mental health needs of the full range of significant others that support mothers, service design will struggle to be inclusive and to support the entire family.

It is important to consider at what point intervention might be most effectively delivered to significant others. In this survey, the mental health of significant others was surveyed at least 1 month after women were discharged from acute services. The prevalence of mental distress in this population may have been even greater during crisis. Future studies could survey mental illness in significant others at the time of admission of mothers to acute care and discharge to indicate how levels might change over time and to quantify the impact of pre-existing illness. Additional research should also survey the willingness of significant others to receive mental health interventions for themselves to ensure the desirability of services.

This study found that 21.2% of significant others to mothers receiving inpatient care reported being in the lowest income group (compared to 4.6% in the CRT group). Though this did not reach statistical significance in our analysis, the income brackets used in the questionnaire did not provide a high level of detail on the financial situation of these families. More specific questions relating to socioeconomic status would be of value in future research.

## Conclusion

This study found that 51% of significant others to women receiving acute postnatal psychiatric care in a variety of settings have clinically significant mental health needs and around 80% of these needs are unmet by current service provision. Meeting the mental health needs of this population is of critical importance to the development and delivery of more inclusive perinatal mental health services and to secure the wellbeing of women, infants, families, and wider support networks.

## Data Availability

Data analysed in this article is part of a wider data set from the ESMI MBU study which has yet to be made available.

## Abbreviations

CRT: Crisis resolution team
GHQ-12: General Health Questionnaire-12
GP: General practitioner
IEQ: Involvement Evaluation Questionnaire
IQR: Interquartile range
MBU: Mother and baby unit
NHS: National Health Service
SD: Standard deviation
UK: United Kingdom
vs.: Versus

## Declarations

### Disclaimer

The views expressed are those of the author(s) and not necessarily those of the NHS, the NIHR, or the Department of Health and Social Care.
